# Routine restaging after primary non-surgical treatment of laryngeal squamous cell carcinoma—a review

**DOI:** 10.1007/s00066-020-01706-9

**Published:** 2020-11-20

**Authors:** Caroline Theresa Seebauer, Berit Hackenberg, Jirka Grosse, Janine Rennert, Ernst-Michael Jung, Ines Ugele, Ioannis Michaelides, Hisham Mehanna, Matthias G. Hautmann, Christopher Bohr, Julian Künzel

**Affiliations:** 1 Department of Otorhinolaryngology, Head and Neck Surgery, University Hospital of Regensburg, Franz-Josef-Strauß-Allee 11, 93053 Regensburg, Germany; 2 Department of Otorhinolaryngology, Head and Neck Surgery, University Medical Center of Mainz, 55131 Mainz, Germany; 3 Department of Nuclear Medicine, University Hospital of Regensburg, 93053 Regensburg, Germany; 4 Institute of Radiology, University Hospital of Regensburg, 93053 Regensburg, Germany; 5 Department of Head and Neck Surgery, University of Birmingham, B15 2TT Birmingham, UK; 6 Department of Radiotherapy, University Hospital of Regensburg, 93053 Regensburg, Germany

**Keywords:** Therapy control, Imaging, Larynx cancer, Organ-preserving treatment, Radiotherapy, Chemoradiotherapy

## Abstract

**Purpose:**

Treatment of patients with laryngeal squamous cell carcinoma with radiotherapy or chemoradiation is an established alternative to laryngeal surgery in many cases, but particularly for advanced tumors without cartilage invasion. Imaging modalities face the challenge of distinguishing between posttherapeutic changes and residual disease in the complex anatomic subsite of the larynx. Guidelines concerning restaging of head and neck squamous cell carcinomas (HNSCC) are presented by the National Comprehensive Cancer Network (NCCN) and other national guidelines, but clearly defined recommendations for routine restaging particularly for laryngeal cancer are lacking.

**Methods:**

A systematic search was carried out in PubMed to identify studies evaluating routine restaging methods after primary non-surgical treatment of laryngeal squamous cell carcinoma from 2009 to 2020.

**Results:**

Only three studies were deemed eligible, as they included at least ≥50% patients with laryngeal squamous cell carcinoma and evaluated imaging modalities to detect residual cancer. The small number of studies in our review suggest restaging with fluoro-deoxy-glucose positron-emission tomography/computed tomography (FDG PET/CT) 3 months after initial treatment, followed by direct laryngoscopy with biopsy of the lesions identified by FDG PET/CT.

**Conclusion:**

Studies evaluating restaging methods after organ-preserving non-surgical treatment of laryngeal carcinoma are limited. As radiotherapy (RT), chemoradiotherapy (CRT), systemic therapy followed by RT and radioimmunotherapy are established alternatives to surgical treatment, particularly in advanced laryngeal cancers, further studies are needed to assess and compare different imaging modalities (e.g. PET/CT, MRI, CT, ultrasound) and clinical diagnostic tools (e.g., video laryngoscopy, direct laryngoscopy) to offer patients safe and efficient restaging strategies. PET or PET/CT 3 months after initial treatment followed by direct laryngoscopy with biopsy of the identified lesions has the potential to reduce the number of unnecessary laryngoscopies.

## Introduction

Laryngeal carcinoma occurs in 3/100,000 persons [1]. Early laryngeal cancers are usually treated by surgery, with favorable results [2, 3]. Currently, patients with advanced laryngeal squamous cell carcinoma (T3, T4) are offered radical surgery (total or near-total laryngectomy) followed by adjuvant therapy or systemic chemoradiation (induction chemotherapy in selected cases) as treatment options [4–9]. Factors like cartilage invasion, voice, swallowing, quality of life, and the patient’s preferences contribute to choosing the appropriate treatment modality [10]. Unfortunately, locally advanced (T3, T4) laryngeal cell carcinomas still show 5‑year overall survival rates of less than 50% [11]. Furthermore, in T2–T4 laryngeal carcinoma, a local or locoregional recurrence rate between 25 and 50% after radiotherapy (RT) or chemoradiotherapy (CRT) is detectable [12]. These numbers emphasize the importance of a structured follow-up for this patient group to detect failure of treatment as early as possible. Planned restaging for tumor response evaluation should be one of the first steps in a patient’s follow-up. Clinical symptoms of residual cancer like pain, dysphagia, hoarseness, and respiratory distress can also occur secondary to radiation toxicity, which complicates diagnosis. Imaging modalities like computed tomography (CT) or magnetic resonance imaging (MRI) sometimes have difficulty in differentiating between posttherapeutic changes (like edema and protracted mucositis/laryngitis) and residual disease, notably in the complex anatomic subsite of the larynx. In general, repeating pretreatment baseline imaging of the primary within 6 months of treatment is recommended for head and neck squamous cell carcinoma (HNSCC). In 2016, de Bree et al. showed that positron-emission tomography/computed tomography (PET/CT) in case of suspected recurrence after (chemo)radiotherapy of laryngeal cancer reduced the number of unnecessary laryngoscopies [13]. However, although guidelines concerning routine restaging of HNSCC are presented by the National Comprehensive Cancer Network (NCCN) and national guidelines, clearly defined recommendations for restaging particularly for laryngeal cancer are missing [14, 15]. Therefore, the objective of this review is to evaluate methods for routine restaging and evaluation of tumor response (partial or complete remission) for laryngeal cancer after treatment with radio- or chemoradiotherapy. We discuss current literature on restaging methods after primary RT, CRT, or radioimmunotherapy of laryngeal squamous cell carcinoma.

## Methods

**Fig. 1:**
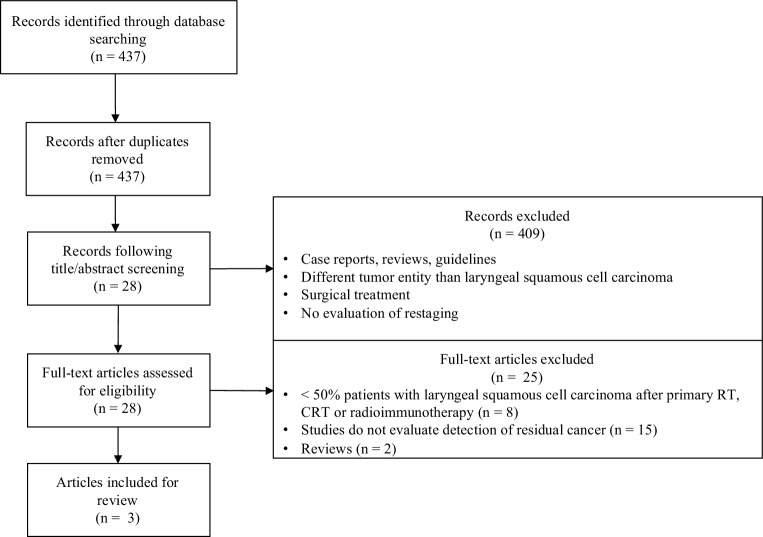
Inclusion and exclusion criteria summarized in a Preferred Reporting Items for Systematic Reviews and Meta-Analyses (PRISMA) flowchart. (*RT* radiotherapy; *CRT* chemoradiotherapy

A systematic literature search of original research articles published in English within the last 10 years (until July 2020) was conducted in PubMed using the search term (laryngeal cancer OR laryngeal carcinoma OR larynx cancer OR larynx carcinoma) AND (radiation therapy OR chemoradiotherapy OR chemoradiation OR radioimmunotherapy) AND (PET OR PET/CT OR PET/MRI OR CT OR MRI OR sonography OR ultrasound OR laryngoscopy OR microlaryngoscopy). The resulting list of articles was screened for duplicates by a public reference manager (Mendeley 1.19.4, Mendeley Ltd, London, UK). Titles and abstracts were screened by three reviewers with regard to the PICO (Patients, Intervention, Comparison, Outcome) framework. Included were only studies with at least ≥50% patients with laryngeal squamous cell carcinoma after primary RT, CRT, or radioimmunotherapy. Only studies evaluating routine restaging strategies were included, studies evaluating diagnostic work-up of suspected recurrence or follow-up studies were excluded. The outcome of interest was detection of residual cancer. Inclusion and exclusion criteria are summarized in the Preferred Reporting Items for Systematic Reviews and Meta-Analyses (PRISMA) flowchart (Fig. 1).

## Results

### Literature search

After applying the filters “<10 years,” “English language,” and “human species,” the PubMed literature search showed 437 references. None had to be excluded due to duplicates. After screening of titles and abstracts, the analysis of 28 full-text articles identified three studies (Table 1) which met the inclusion criteria of the review. 409 studies were excluded because they were case reports, reviews, clinical guidelines, tumor entities other than squamous cell carcinoma of the larynx, received surgical treatments, and/or did not evaluate restaging techniques. On screening full-text articles, eight studies were excluded as they included less than 50% of patients with laryngeal cancer in their cohort. 15 studies were excluded because they did not evaluate the detection of residual cancer after primary RT, CRT, or radioimmunotherapy. Two studies appeared to be reviews of literature and not original research articles when screening the full-text articles. Data analysis was performed to compare restaging methods after organ-preserving treatment of laryngeal carcinoma with RT, CRT, or radioimmunotherapy regarding their diagnostic value. Therefore, the following information was extracted from studies analyzed: name of first author, publication date, type of study, patient number, tumor stage, tumor localization, treatment information, time to imaging after therapy, posttherapeutic imaging modalities, sensitivity, specificity, accuracy, positive predictive values, negative predictive values, and median follow-up time (Table 1). In all of the studies listed in Table 1, confirmation of residual tumor was obtained by imaging follow-up and/or histological examination.

**Table 1:** Clinical studies on restaging modalities of laryngeal squamous cell carcinoma after radiotherapy, chemoradiotherapy, or radioimmunotherapy

First author	Year	Type of study	Patient number T stage (*n*, %)	Laryngeal subsite (*n*, %)			Treatment (*n*, %)	RT (*n*, %)	STH	Time to imaging after therapy	Posttherapeutic imaging (*n*)	Sensitivity	Specificity	Accuracy	PPV	NPV	FU
Been [16]	2009	Prospective	–	*Glottis*	*Supraglottis*	*Subglottis*	–	–	–	–	–	–	–	–	–	–	–
			*14*	11, 79%	2, 14%	1, 7%	RT 14, 100%	70 Gy	None	2 mo–3 mo	FDG PET 10^a^	67%	86%	80%	67%	86%	24 mo
				T1 4, 29%	T1 1, 7%	T1 0											
			T1 5, 36%	T2 5, 36%	T2 0	T2 0		C 14, 100%									
			T2 5, 36%	T3–T4 1, 7%	T3 1, 7%	T3 0	CRT 0	IMRT 0			FLT PET 10^a^	33%	100%	80%	100%	78%	
			T3–T4 4, 29%	T4 1, 7%	T4 0	T4 1, 7%											
Mayo [17]	2019	Retrospective	*28*	8, 29%	20, 71%	0	RT 1, 4%	70–80 Gy	Cisplatin	3 mo	FDG PET/CT 26^b^	33%	85%	73%	40%	81%	35 mo
			T1 0														
			T2 0					C 28, 100%	Cisplatin/carboplatin with paclitaxel								
			T3 25, 89%	n.a.	n.a.	n.a.	CRT 27, 96%										
			T4 3, 11%					IMRT 0	Cetuximab								
Wedman [18]	2018	Prospective	*47*	20, 43%	24, 51%	3, 6%	RT 44, 94%	n.a.	n.a.	3 mo–12 mo	FDG PET 26^c^	75%	53%	n.a.	62%	73%	24 mo
			T1 0														
			T2 26, 55%														
			T3 17, 36%	n.a.	n.a.	n.a.	CRT 3, 6%				MET PET 26^c^	53%	76%	n.a.	65%	63%	
			T4 4, 9%														

*C* conventional, *CT* computerized tomography, *CRT* chemoradiotherapy, *FDG PET* [18F]-2-fluoro-2-deoxy-D-glucose ([18F]FDG) positron-emission tomography, *FLT PET* [18F]-fluoro-30-deoxy-L-thymidine ([18F] FLT) positron-emission tomography, *FU* follow-up, *IMRT* intensity-modulated radiotherapy, *MET* C-11 methionine, *mo* months, *n.a.* not available, *NPV* negative predictive value, *PPV* positive predictive value, *RT* radiotherapy, *STH* systemic therapy, *T* tumor stage

^a^10 patients underwent all four PET scans, 4 patients only underwent the pretherapeutic scans

^b^26 patients with local residual tumor, 2 patients with regional or distant tumor recurrence

^c^26 patients included between 3 and 12 months after finishing radiotherapy. The rest of the patients were included between 1 and 5 years after finishing therapy and imaging was therefore not performed with regard to restaging

### Findings from clinical studies

Table 1 summarizes three articles with regard to restaging methods after organ-preserving treatment of laryngeal carcinoma with RT or CRT. In the above-mentioned studies, none of the patients were treated with radioimmunotherapy. Overall restaging modalities were evaluated in 89 patients, of whom 53 (59.6%) had advanced laryngeal cancer (tumor stage T3, T3–T4, T4). 59 (66.3%) patients were treated with RT, 30 (33.7%) with CRT. One study obtained data retrospectively with 28 patients included in the study, whereas two studies were prospective with 47 and 14 evaluated patients. The time to imaging after RT or CRT varied from 2 to 12 months. The median time for which the patients were followed up was 24 to 35 months.

The restaging methods discussed in those studies comprise [18F]-2-fluoro-2-deoxy-D-glucose ([18F] FDG) positron-emission tomography (FDG PET), [18F]-fluoro-30-deoxy-L-thymidine ([18F] FLT) positron-emission tomography (FLT PET), and C‑11 Methionine (MET) PET. FDG is the most widely used radioactive PET tracer in oncology. Drawbacks of FDG are the physiological uptake in muscles of the larynx and the uptake by inflammatory or reactive tissues occurring after radiotherapy. The sensitivity of FDG PET to detect residual tumor after RT or CRT was 33% in one study [17], and 67 and 75% in two other studies [16, 18]. Been et al. and Mayo et al. reported a specificity of 86 and 85% for FDG PET, respectively [16, 17], whereas Wedman et al. showed a lower specificity of 53% [18]. The PPV in the study by Mayo et al. for FDG PET was 40% [17], which was lower compared to the PPV of 67 and 62% detected in the studies of Been et al. and Wedman et al., respectively [16, 18]. All three studies showed a comparable NPV, with 86, 81, and 73%, respectively [16–18]. All studies investigated imaging 2–12 months after finishing radiotherapy. In the study of Wedman et al., 17 patients were included between 1 and 5 years after finishing therapy. Therefore, imaging was not performed with regard to restaging and the results of these patients are not included in this review. Been et al. included FLT PET as a restaging modality in their study and found a sensitivity of 33% and a specificity of 100%. FLT is phosphorylated by thymidine kinase 1 and trapped in the cell. Its activity is increased in proliferating cells like malignant cancer cells. After radiotherapy, the sensitivity of FDG to detect residual tumor was higher as compared to FLT [16]. Wedman et al. also described MET PET as a restaging method, which had a sensitivity of 53% and a specificity of 76%. C‑11 MET is an established radiopharmaceutical and has been successfully used for visualizing primary head and neck cancer. The uptake of amino acids is high in tumor cells but low in inflammatory tissues and could therefore be a good alternative to distinguish residual tumor cells from posttherapeutic inflammation after radiotherapy. The PPV of MET was not significantly higher than the PPV obtained with FDG. Therefore, this study implies that MET PET cannot be used to select patients for a direct laryngoscopy compared to FDG PET.

## Discussion

Currently, the American National Comprehensive Cancer Network (NCCN) clinical practice guidelines in oncology for head and neck cancers (version 3.2019–September 16, 2019) [19] recommend a clinical assessment 4–8 weeks after treatment of laryngeal carcinoma with CRT or RT alone. If the clinical assessment shows tumor response, a CT of the primary cancer site and the neck and/or an MRI with contrast should be performed within 8–12 weeks or an FDG PET/CT should be carried out within 12 weeks to assess the extent of the disease. In case the clinical assessment suggests residual primary tumor, persistent disease, or disease progression, a CT and/or an MRI with contrast within 4–8 weeks or an FDG PET/CT should be considered. Accurate diagnostic tools are important to ensure that the correct treatment recommendations are made [20]. Therefore, sensitivity, specificity, accuracy, PPV, and NPV are important parameters to determine the optimal restaging modality for patients with laryngeal squamous cell carcinoma who have been treated by RT or CRT.

The current 2A recommendations from the NCCN are based on a review by Kutler et al. [21] evaluating the role of neck dissection following definitive CRT [21]. Restaging methods particularly for laryngeal cancer treated with induction chemotherapy followed by RT, RT, alone or CRT are lacking. Despite this, in recent decades, refinements in radiotherapy technique and protocols for induction chemotherapy have made non-surgical organ-preserving treatment methods for laryngeal cancer an established alternative to laryngectomy [7, 22].

Focusing the literature search on restaging methods after RT, CRT, or radioimmunotherapy of laryngeal squamous cell carcinoma limited the evaluated restaging methods to FDG PET/CT (Table 1). Other publications evaluated in the full-text screening analyzed restaging methods after RT, CRT, or radioimmunotherapy for patients with HNSCC, including other tumor locations. Those studies examined computerized tomography (CT), magnetic resonance imaging (MRI), diffusion-weighted MRI (DW MRI), outpatient video laryngoscopy with white light and narrow-band imaging (NBI), and direct laryngoscopy with white light and NBI, in addition to FDG PET/CT and direct laryngoscopy. These studies were excluded as they included less than 50% of patients diagnosed with laryngeal cancer or evaluated imaging modalities that detect recurrent tumor lesions rather than restaging methods.

In a heterogenous HNSCC patient cohort, sensitivity for detecting recurrent or residual tumor after RT or CRT was determined for direct laryngoscopy under general anesthesia with white light and NBI (100%) in 68 patients, compared to 88% in outpatient video laryngoscopy with white light and NBI in 66 patients [23]. Next, DW MRI showed a sensitivity of 94% in a study with 46 patients [24] and a sensitivity of 69% in an article evaluating 70 patients [25]. Another study investigating the use of dynamic contrast-enhanced MRI in predicting early response to CRT in HNSCC patients found a sensitivity and specificity of 89.3 and 73.5%, respectively [26]. The sensitivity for MRI was 72% in a study analyzing 46 patients [24], whereas CT showed a sensitivity of 68% in a study with 111 patients [27]. The most specific restaging modalities were outpatient video laryngoscopy with white light and NBI, as well as direct laryngoscopy with white light and NBI (both 92%) [23]. The specificity of DW MRI varied between 100% [24] and 77% [25], whereas CT demonstrated a higher specificity (88%) [27] than MRI (57%) [24]. The PPV for DW MRI was either 100% [24] or 75% [25], followed by 79% for outpatient video laryngoscopy with white light and NBI and direct laryngoscopy with white light and NBI [23]. The lowest PPV were evaluated for MRI (52%) [24] and CT (50%) [27]. NPV were especially high in outpatient video laryngoscopy with white light and NBI and direct laryngoscopy with white light and NBI (96 and 100%) [23]. CT (8/93%) [27] showed similar NPV to DW MRI (71% [25] and 97% [24]), followed by MRI with 76% [24].

Other authors have investigated the use of FDG PET/CT in restaging after HNSCC [25, 27–32]. The patient cohorts were not focused on laryngeal carcinoma. To our knowledge, the three articles analyzed in this review are the only publications focusing on restaging of laryngeal carcinoma after RT, CRT, or radioimmunotherapy in the past 10 years. For the detection of residual or recurrent HNSCC, the sensitivity of FDG PET/CT ranges from 71 to 97%, whereas specificity ranges from 46 to 92%, the PPV ranges from 64 to 71%, and the NPV from 86 to 98% [25, 27, 31, 33]. Analyzing the studies in Table 1, the sensitivity of FDG PET/CT for detecting residual laryngeal carcinoma ranges from 33 to 75%, specificity from 53to 86%, PPV from 40 to 67%, and NPV from 73 to 86%.

Literature on the value of FDG PET/CT imaging in the diagnosis and staging of patients with laryngeal carcinoma shows a higher sensitivity (100%) than MRI/CT (93.3%); additionally, FDG PET/CT is able to detect regional nodal and distant metastasis, as well as synchronous tumors [34]. Reasons for the lower accuracy of FDG PET/CT in restaging of laryngeal carcinoma after organ-preserving non-surgical treatment may be false-positive results due to inflammation, edema, or protracted mucositis/laryngitis, which increase the necessity of laryngoscopies with biopsies under general anesthesia. It is important to note that several studies have demonstrated that FDG PET/CT is less sensitive early after treatment and is best carried out 12 weeks post CRT to minimize the risk of false-positive results [19, 35–37]. In our review, the time between therapy and imaging with FDG PET/CT was 2–12 months. Other issues with FDG PET/CT are the high costs and availability of the procedure. An analysis of Smith et al. published in 2016 indicates that PET/CT-guided patient management is cost effective in the long-term in a UK-based patient cohort [38]. Another possible restaging method is the use of FLT or MET instead of FDG in positron-emission tomography. These methods showed lower sensitivities and NPV, but higher values for specificity and PPV. The overall tracer uptake was significantly lower as compared to FDG, and tumor-to-background ratios were comparable or lower than the ratio obtained with FDG [16, 18]. Still, the authors deemed both modalities feasible for visualizing laryngeal cancer.

Positive results in restaging with FDG PET/CT need to be confirmed by biopsy during a laryngoscopy under general anesthesia. In the study of de Bree et al., laryngoscopy without previous imaging showed a PPV of only 32% [13] due to a high number of false-positive results, which in this study equaled an unnecessary indication for a laryngoscopy. Zabrodsky et al. found a PPV of 79% for direct laryngoscopy when combined with narrow-band imaging [23]. Outpatient video laryngoscopy with narrow-band imaging showed the same PPV (79%) and specificity (92%) as direct laryngoscopy, but the sensitivity was higher in direct laryngoscopy (100%) compared to video laryngoscopy (88%) [23] and equal to a study by Terhaard et al. published in 2001 [39].

When looking into restaging laryngeal cancer with CT or MRI after treatment with systemic therapy and/or radiotherapy, our literature research did not reveal any studies published within the past 10 years. With the mentioned low PPV for MRI (52%) [24] and CT (50%) [27], both modalities cannot reliably differentiate residual or recurrent disease from postirradiation changes in laryngeal carcinoma, which has been demonstrated previously by other reviews [40].

Ultrasound, another important diagnostic tool [41], was also not evaluated by any study in the past 10 years looking at restaging of laryngeal cancer treated with RT or CRT. A prospective study by de Fiori et al. published in 2016 demonstrated a sensitivity of 85.7% and a specificity of 100% for ultrasound-guided transcutaneous biopsy in 19 patients with laryngeal and hypopharyngeal carcinoma [42]. Contrast-enhanced ultrasound already plays a role in therapy control and monitoring of liver tumors and could at least be helpful to support treatment response evaluation of cervical lymph nodes [43, 44]. Endoscopic ultrasound offers high-resolution imaging of endolaryngeal structures and their pathological changes [45]. Further studies are needed to evaluate the role of ultrasound in restaging after organ-preserving non-surgical treatment of laryngeal carcinoma.

Overall, FDG PET/CT in combination with direct laryngoscopy seems to be the most favorable restaging method to assess laryngeal squamous cell carcinoma after treatment with RT or CRT. With Mehanna et al. demonstrating that FDG PET/CT 12 weeks after primary non-surgical treatment can reduce the necessity of salvage neck dissection and de Bree et al. showing that FDG PET/CT in restaging lowers the need for direct laryngoscopies under general anesthesia, insurance policies are willing to cover the costs of the procedure [13, 46, 47]. To avoid false-positive results, but still remain eligible for surgical treatment, FDG PET/CT should be performed 12 weeks after the end of primary non-surgical treatment [48]. Finally, there is a need for many more prospective studies evaluating the restaging modalities, especially FDG PET/CT and DW MRI, for laryngeal squamous cell carcinoma after systemic therapy with radiotherapy, CRT, or RT alone [49].

## Conclusion

**Fig. 2:**
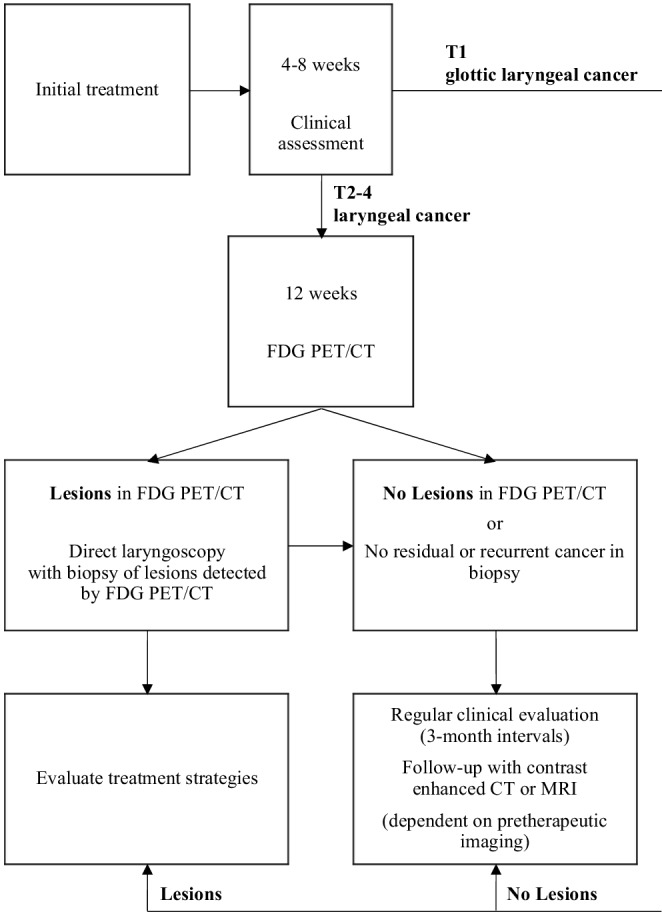
Restaging after primary non-surgical treatment of laryngeal squamous cell carcinoma

Studies evaluating restaging methods after organ-preserving non-surgical treatment of laryngeal carcinoma are limited. As RT, CRT, and systemic therapy followed by RT are established alternatives for surgical treatment particularly in advanced laryngeal cancers without cartilage invasion, further prospective studies are needed to assess and compare different imaging modalities (e.g., FDG PET/CT, MRI, CT, ultrasound) and clinical diagnostic tools (e.g., video laryngoscopy, direct laryngoscopy with NBI) to offer patients safe and efficient restaging strategies. The small number of studies in our review does not allow for clear suggestions regarding restaging of laryngeal cancer. However, looking at the available data and guidelines, FDG PET/CT 3 months after initial treatment followed by direct laryngoscopy with biopsy of the lesions identified by FDG PET/CT is a reasonable approach for T2–T4 laryngeal carcinomas (Fig. 2). This procedure has the potential to reduce the number of unnecessary laryngoscopies. T1 glottic laryngeal cancers usually allow solely clinical evaluation due to their location and size. After a negative FDG PET/CT or unremarkable laryngoscopy with biopsy, regular clinical evaluation with annual repetition of the pretherapeutic imaging is a reasonable follow-up strategy. The development of clinical guidelines specifically for restaging of laryngeal carcinoma after non-surgical treatment is necessary.
